# Conditions for success of engineered underdominance gene drive systems

**DOI:** 10.1016/j.jtbi.2017.07.014

**Published:** 2017-10-07

**Authors:** Matthew P. Edgington, Luke S. Alphey

**Affiliations:** The Pirbright Institute, Ash Road, Woking, Surrey, GU24 0NF, UK

## Abstract

Engineered underdominance is one of a number of different gene drive strategies that have been proposed for the genetic control of insect vectors of disease. Here we model a two-locus engineered underdominance based gene drive system that is based on the concept of mutually suppressing lethals. In such a system two genetic constructs are introduced, each possessing a lethal element and a suppressor of the lethal at the other locus. Specifically, we formulate and analyse a population genetics model of this system to assess when different combinations of release strategies (i.e. single or multiple releases of both sexes or males only) and genetic systems (i.e. bisex lethal or female-specific lethal elements and different strengths of suppressors) will give population replacement or fail to do so. We anticipate that results presented here will inform the future design of engineered underdominance gene drive systems as well as providing a point of reference regarding release strategies for those looking to test such a system. Our discussion is framed in the context of genetic control of insect vectors of disease. One of several serious threats in this context are *Aedes aegypti* mosquitoes as they are the primary vectors of dengue viruses. However, results are also applicable to *Ae. aegypti* as vectors of Zika, yellow fever and chikungunya viruses and also to the control of a number of other insect species and thereby of insect-vectored pathogens.

## Highlights

• A model of engineered underdominance (UD) gene drive is proposed.
• Numerical results suggest that UD could be an effective tool for disease control.
• Success/failure of UD is affected by release strategy and fitness of mosquitoes.
• A single release of only males with weakly suppressed lethals cannot succeed.

## 1 Introduction

*Aedes aegypti* mosquitoes are the primary vector of dengue viruses (World Health Organization, 2016a). These are a great concern for public health burden in over one hundred countries, mainly located in tropical and sub-tropical regions (Bhatt, Gething, Brady, Messina, Farlow, Moyes, Drake, Brownstein, Hoen, Sankoh, Myers, George, Jaenisch, Wint, Simmons, Scott, Farrar, Hay, 2013, Brady, Gething, Bhatt, Messina, Brownstein, Hoen, Moyes, Farlow, Scott, Hay, 2012). There are more than two billion people living in at risk areas and tens of millions of apparent infections are estimated to occur each year (Bhatt et al., 2013). This is a particularly serious threat since there are no known drug treatments available (World Health Organization, 2016a) and the first vaccine has only recently been licensed for use (Pitisuttithum and Bouckenooghe, 2016) but is only recommended in areas with a high dengue burden and only in those over nine years of age (World Health Organization, 2016b). There are also a range of other insect-vectored pathogens that pose serious threats to public health including Zika, malaria and filariasis. Current methods do not appear sufficient to deal with these problems (Burt, 2014) suggesting a necessity to investigate alternate methods of control.

In recent years there have been rapid advances in tools available to molecular biologists. This has made the idea of genetic control methods a very real prospect. As such, a number of different genetic control strategies have been proposed that could either supplement or replace the methods currently in place (Alphey, 2014). One category of genetic control, would be introduced by releasing into the wild, insects carrying modified genes rendering them refractory to one or more pathogens of medical importance, such as one or more dengue viruses (i.e. they have a greatly reduced capacity to infect humans). These modified genes would be combined with a gene drive mechanism, causing them to be inherited by the progeny of released mosquitoes at a super-Mendelian rate, meaning they could spread towards fixation in a population over the course of a number of generations (Champer et al., 2016).

**Fig. 1:**
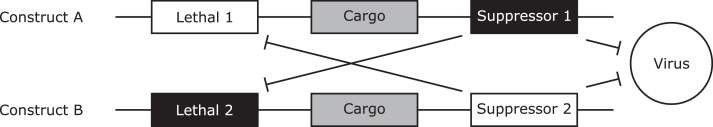
A schematic diagram of a two-locus engineered underdominance gene drive system. Here each of the two genetic constructs consists of a lethal element, a cargo gene and a suppressor for the lethal at the other locus. In such a system an individual will survive if they possess none of the constructs (i.e. they are wild-type) or if they possess at least one copy of both, but die if they carry only one. This creates an appropriate fitness differential between genotypes leading to this being termed an underdominant system. It is assumed here that the components of each transgenic construct cannot separate from one another (methods to mitigate this issue for a *Medea* gene drive system were discussed by Hay et al., 2010).

One such class of gene drive system that is currently under development is known as engineered underdominance. Underdominance, in a single locus setting, refers to the situation whereby a heterozygous individual is less fit than homozygous individuals. Such single-locus underdominance represents a gene drive system in its own right and has been engineered in *Drosophila melanogaster* (Reeves et al., 2014) and also considered theoretically (Altrock, Traulsen, Reeves, Reed, 2010, Altrock, Traulsen, Reed, 2011). In the example considered here, a similar effect is achieved via the introduction of two independently inherited transgenic constructs each carrying a lethal genetic element and a suppressor for the lethal at the other locus (Fig. 1) (Davis et al., 2001). In essence this can be thought of as two killer-rescue (Gould et al., 2008) systems split across two transgenic constructs. Linked to each of the transgenic constructs are cargo genes, here assumed to be genes rendering individuals refractory to dengue, for example those developed by Franz and colleagues (Franz, Sanchez-Vargas, Adelman, Blair, Beaty, James, Olson, 2006, Franz, Sanchez-Vargas, Raban, Black IV, James, Olson, 2014). In this type of system, individuals survive only if they carry no transgenic constructs or if they carry at least one copy of both. This creates a selection pressure for individuals to carry both transgenic constructs and thus potentially allows the refractory genes to be spread throughout a population. If these genes spread to fixation within a population, we would expect that over time infections with the targeted dengue strain(s) would be eliminated, or at least significantly reduced, in the affected area.

Two of the most important classifiers of gene drive systems are related to their persistence and invasiveness. Persistence can be thought of in terms of two distinct categories; self-limiting systems where transgenes naturally fade away over time or self-sustaining systems where transgenes persist indefinitely in the absence of a genetic change (i.e. mutation) and may even increase in prevalence over time (Alphey, 2014). Previous theoretical studies of engineered underdominance systems have demonstrated that they sit on the borderline between persistence classifications (Davis, Bax, Grewe, 2001, Magori, Gould, 2006). In particular, such systems have a threshold for the introduction of insects above which transgenes will spread (self-sustaining) and below this the transgenes will be eliminated (self-limiting). Mathematically this can be thought of as three equilibrium points; two stable equilibria typically representing fixation of wild-type or introduced alleles and an unstable equilibrium that determines which allele heads toward fixation (Magori and Gould, 2006). The exact size of this introduction threshold (unstable equilibrium) in terms of allele frequencies is dependent on the fitness of transgenic insects relative to wild-type individuals (Magori and Gould, 2006). In terms of invasiveness, a gene drive may either be defined as ‘global’ where it would be expected to spread into every insect in every linked population or ‘local’ whereby there would only be spread within a target population (i.e. that into which transgenic insects are introduced). The invasiveness of gene drive systems has been a key consideration in terms of their regulation (Oye et al., 2014) as the Cartagena Protocol prohibits the release of any system capable of spreading across international borders, unless a prior agreement has been reached (Marshall, 2010). Invasiveness is also a key ecological consideration since precise interventions will potentially allow effects to a target pest species with little or no impact on other populations (Hay et al., 2010). As such, it could be considered desirable for a gene drive to spread within a target population but not beyond. Previous theoretical work has shown that engineered underdominance systems are extremely unlikely to spread to fixation in any non-target populations (i.e. those linked to the target population via migration) as the introduction threshold is too large to be reached through migration alone (Marshall and Hay, 2012). This same work went on to show that typical migration rates may only result in  ∼ 3.2% of the non-target population being made up of transgenic mosquitoes. Thus, engineered underdominance appears to be an exciting prospect in that these systems are feasible to engineer and seem able to satisfy a number of key regulatory and ecological issues associated with the release of transgenic insects.

Previous theoretical work on engineered underdominance systems (Buchman, Ivy, Marshall, Akbari, Hay, Davis, Bax, Grewe, 2001, Magori, Gould, 2006, Marshall, Hay, 2012) has focused on the case whereby transgenic individuals of both sexes are released into the wild population; carry lethals that affect both sexes; and display full suppression of one or two copies of a given lethal depending upon the number of copies of the relevant lethal suppressor possessed. Whilst these assumptions are reasonable, there are a number of other possible release strategies (number, size and sex of releases) and genetic systems (sex specificity of lethals and strengths of lethal suppression) that are yet to be considered. Here, we formulate a population genetics model of the engineered underdominance system in Fig. 1 that is capable of representing a range of these possible release strategies and genetic systems. We then use this model to investigate a number of different examples in terms of the restrictions that each will place on the release ratio and fitness costs (measured in terms of reduced survival) of carrying transgenic constructs that may be tolerated by each system while giving transgene introgression. It is then possible to identify the most feasible systems/strategies for real-world deployment along with those which may prove more difficult. It is anticipated that this study will inform future work seeking to develop engineered underdominance gene drive systems and be of interest to those planning to test a particular system/strategy ready for deployment in the field.

## 2 Methods

### 2.1 Mathematical model

We present a population genetics model describing the two-locus engineered underdominance based gene drive system shown in Fig. 1 (see Appendix A for details). This deterministic model is similar to other published models in that it assumes an infinite, closed, panmictic (randomly mating) population with discrete, non-overlapping generations and a 1:1 male to female ratio in both the initial population and eggs laid in subsequent generations (Davis, Bax, Grewe, 2001, Magori, Gould, 2006, Marshall, Hay, 2012). This allows a number of factors to be eliminated including migration between populations. Further, it is assumed that transgenic constructs are not sex linked; no resistance will evolve; transgenic constructs do not mutate; and the cargo and suppressor genes are perfectly linked (i.e. they cannot separate; the effects of which have been modelled for a transposon based gene drive system (Marshall, 2008)) within the modelled population. This results in a model that considers the frequencies of nine distinct genotypes, denoted here by the presence (*A* or *B*) or absence (*a* or *b*) of transgenic constructs A and B, respectively (see Table 1 for details).

**Table 1:** A table summarising the genotype notation used throughout this study. A two-locus engineered underdominance system creates nine possible genotypes. Each is assigned a genotype number (*i*) within the model and are described by the presence (*A* or *B*) or absence (*a* or *b*) of transgenic constructs A and B, respectively. Within the model the numbers of each transgenic construct carried by a particular genotype (*i*) are denoted $\eta_{A}^{i}$ and $\eta_{B}^{i}$ for transgenic constructs A and B, respectively.

*i*	Genotype	Number of Transgenic Constructs Carried	
		$\eta_{A}^{i}$	$\eta_{B}^{i}$
1	*aabb* (wild-type)	0	0
2	*aaBb*	0	1
3	*aaBB*	0	2
4	*Aabb*	1	0
5	*AaBb*	1	1
6	*AaBB*	1	2
7	*AAbb*	2	0
8	*AABb*	2	1
9	*AABB* (introduced)	2	2

In subsequent sections we examine the effects of the relative fitness of individuals carrying transgenic constructs; lethal elements acting on different sexes; and different strengths of lethal element suppression. Rather than formulate a new model in each case, these effects are captured in the model by appropriate choices of parameter values with the precise configurations used in each case discussed within the relevant sections. A summary of all variables and parameters is given in Table 2.

**Table 2:** A table of definitions and constraints for each parameter and variable used within the model.

Symbol	Description	Constraint/Expression
$M_{i}^{t}$	Proportion of males of genotype *i* in	$\sum_{i=1}^{9}\left(M_{i}^{t}+F_{i}^{t}\right)=1$
	generation *t* (normalised)	and $0\leq M_{i}^{t}\leq 1$
$F_{i}^{t}$	Proportion of females of genotype *i* in	$\sum_{i=1}^{9}\left(M_{i}^{t}+F_{i}^{t}\right)=1$
	generation *t* (normalised)	and $0\leq F_{i}^{t}\leq 1$
$M_{i}^{e}$	Proportion of males of genotype *i* in	–
	generation *t*	
$F_{i}^{e}$	Proportion of females of genotype *i* in	–
	generation *t*	
$\bar{\Omega }$	Average fitness of whole population in the	$\sum_{i=1}^{9}\left(M_{i}^{e}+F_{i}^{e}\right)$
	current generation	
ϵ_*A*_	Relative fitness of individuals with one copy	0 ≤ ϵ_*A*_ ≤ 1
	of construct A	
ϵ_*B*_	Relative fitness of individuals with one copy	0 ≤ ϵ_*B*_ ≤ 1
	of construct B	
$\eta_{i}^{A}$	Number of copies of construct A carried by an	$\eta_{i}^{A}=0,1,2$
	individual of genotype *i*	
$\eta_{i}^{B}$	Number of copies of construct B carried by an	$\eta_{i}^{B}=0,1,2$
	individual of genotype *i*	
$\gamma_{i}^{M,F}$	Lethality to males (*M*) or females (*F*)	$0\leq \gamma_{i}^{M,F}\leq 1$
	due to presence of constructs in individuals	
	of genotype *i*	
$\Omega_{i}^{M,F}$	Total relative fitness of males (*M*) or	$\Omega_{i}^{M,F}=\epsilon_{A}^{\eta_{i}^{A}}\epsilon_{B}^{\eta_{i}^{B}}\left(1-\gamma_{i}^{M,F}\right)$
	females (*F*) of genotype *i* possessing	
	copies of the constructs, including lethality	
	in the current generation	
*α*	Release ratio (*α*:1, introduced:wild)	α=Introduced/Wild-Type

### 2.2 Investigating effects of parameters

In some of the subsequent sections the effects of different parameter values are assessed. Rather than attempting to infer the pattern of behaviour from simulations using a number of specifically chosen parameter sets, here the entire biologically feasible range is tested. In order to do this a numerical script was created in Matlab (The MathWorks Inc., Natick, MA) which discretises the biologically feasible parameter space into a grid of points each representing a particular parameter configuration. Numerical simulations are conducted for each of these points and are continued for a sufficient number of generations to approach equilibrium. The equilibrium frequencies of relevant genotypes are then stored in an array allowing the equilibria over the entire parameter range to be visualised.

### 2.3 Calculating the number of generations to equilibrium

Whilst the main focus of this study is on evaluating what the final outcome of a given genetic control system will be under certain parameter sets, it is also important to assess systems/strategies against other measures. One criterion that has been stated as key in the evaluation of genetic control methods is that any control strategy should be effective “within a human, not evolutionary time frame” (James, 2005). This essentially means that a genetic control strategy should achieve its desired goal while allowing humans to observe its effects and in a time period over which it could be practical to provide the relevant resources. In order to assess whether this is true of the various configurations of engineered underdominance considered here, each single release case is assessed in terms of the number of generations taken for the wild-type population to be replaced. This is done in a similar manner to evaluating parameter effects in that it is assessed over a discretised grid of parameter configurations. However, in this case the number of generations taken until the wild-type (*aabb*) genotype frequency gets close to zero and stays there (i.e. falls below 0.05 and changes by less than 0.0001 between two consecutive generations) are obtained and visualised. A wild type proportion of  < 5% of the total population is assumed to be a reasonable threshold for the elimination of viruses such as dengue based on the work of (Focks and Barrera, 2006). We consider only the wild-type proportion as important since they are the only genotype able to transmit dengue (assuming that the cargo is effective in a single copy).

## 3 Single release strategies

### 3.1 Effects of different releases, lethals and suppressors

Whilst the general principle of two mutually repressing lethal genetic constructs is well established, there has been relatively little discussion around the types of lethal and suppressor components that could be used. The different options available are numerous and may lead to significantly different outcomes for the same release strategy. In particular, there are known lethal genetic elements in a number of species that will affect either both sexes (bisex lethals) or females only (Fu, Condon, Epton, Gong, Jin, Condon, Morrison, Dafa’alla, Alphey, 2007, Heinrich, Scott, 2000, Thomas, Donnelly, Wood, Alphey, 2000) and a number of these have been modelled (Akbari, Matzen, Marshall, Huang, Ward, Hay, 2013, Marshall, 2011). There could also be lethal elements targeting only males. Male-lethal systems are anticipated to show broadly similar dynamics to female-lethal systems. One exception is a reduced degree of suppression of the vector population during spread, both because increased male mortality likely has less effect on population reproductive potential than female mortality. Male-killing systems are not further considered here.

It has also been demonstrated that lethal elements can be suppressed with varying degrees of effectiveness. Previous theoretical work has focused on systems in which one copy of a suppressor gene is sufficient to rescue an individual against two copies of a lethal gene (from here on referred to as “strongly suppressed lethals”) (Davis, Bax, Grewe, 2001, Huang, Lloyd, Legros, Gould, 2009, Magori, Gould, 2006, Marshall, Hay, 2012). This differs from the work of  Buchman et al. (2016) who modelled and engineered in *Drosophila melanogaster* a system of chromosomal translocations in which suppression is less effective, requiring equal copies to rescue against the lethal effect (“weakly suppressed lethals”).

In addition to this, two distinct release strategies have been discussed for other gene drive systems. The first of these involves the release of both sexes (here “bisex releases”) while the other is based on the release of males only (Alphey and Bonsall, 2014b). For a similar reason to the lethal components, consideration of female-only releases is not pursued any further since the addition of extra females into a population would at the very least increase the rate of nuisance biting and might contribute to population growth. This would be counter-productive in terms of the public perception of any control programme and possibly create adverse effects on disease prevalence and so has not been considered a preferred approach for the genetic control of insects.

**Fig. 2:**
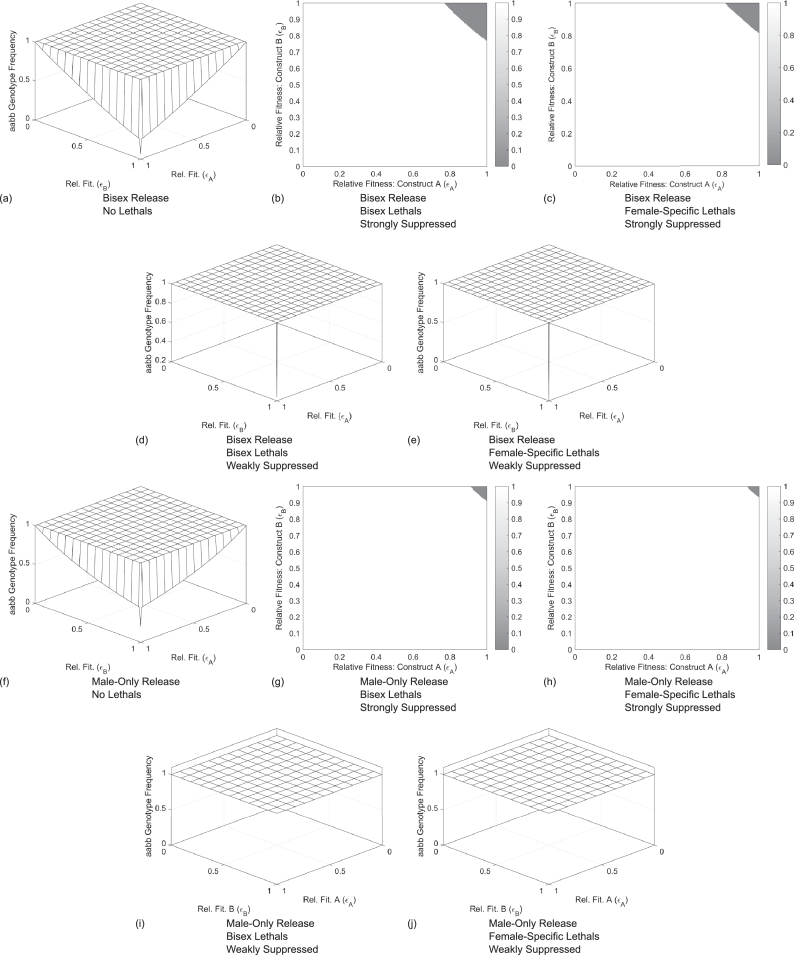
Plots showing wild-type (*aabb*) equilibrium genotype frequencies for different single release strategies and genetic systems. Each plot shown here is for a 1:1 (introduced to wild-type) release ratio. Here three different types of lethality are considered, namely no lethals, bisex lethals and female-specific lethals. This is in addition to consideration of two different strengths of suppressor elements termed a strong and a weak suppressor. Finally, both bisex and male-only releases are considered. For cases with either no lethal elements or weakly suppressed lethals it is clear that the transgenic constructs must confer no fitness cost (i.e. ${ɛ}_{A}=1={ɛ}_{B}$) in order to achieve any degree of lasting transgene introgression. This is in contrast to those with strongly suppressed lethal elements which allow some fitness cost (i.e. ε_*i*_ ≤ 1 for *i* ∈ {*A, B*}) and still achieve full transgene introgression. Some panels are represented on three dimensional axes to aid visualisation.

Within this section the potential outcomes of a number of different control strategies are examined. These are then compared to the introduction of two genetic constructs that have no lethal genes but simply confer some fitness cost on an individual due to their presence in the genome. To make an equal comparison between the different strategies/systems, a single release at a ratio of 1:1 (released transgenic adults to total wild-type adults of both sexes) is modelled for each. This leads to the consideration of two different sets of initial conditions representing bisex and male-only releases, respectively (see Appendix A for details). Then using the method described in Section 2.2, the outcome of each control strategy is evaluated by considering the wild-type frequency at equilibrium for a range of different relative fitness parameters associated with the carrying of transgenic constructs. Results of this are given in Fig. 2. Note that the case of a bisex release of individuals carrying weakly suppressed bisex lethals is very similar to the chromosomal translocation system studied by  Buchman et al. (2016) and will also display similar behaviour to single locus underdominance systems such as that developed by  Reeves et al. (2014).

It is clear from Fig. 2(a) and (f) that the introduction of transgenic constructs conferring a fitness cost but no lethal component will not lead to any degree of long-term introgression for a 1:1 release ratio. However, if these constructs were able to be inserted without a fitness cost (i.e. ${ɛ}_{A}=1,$${ɛ}_{B}=1$ or both) then there would be no selection pressure for the transgenes to be eliminated. Thus they would remain in the population at the initial transgene frequency (although this may be divided across multiple genotypes). It is also expected that introducing transgenic constructs with no lethal elements could possibly lead to some degree of persistence where there is a small fitness cost associated with carrying them, however they still reduce in frequency at some rate depending on the size of the fitness cost.

Similar to the examples with no lethal elements, strategies based on 1:1 releases with weakly suppressed lethals (Fig. 2(d), (e), (i) and (j)) fail to give introgression where the constructs confer even a very small fitness cost. In the examples based on a single 1:1 bisex release (Fig. 2(d) and (e)) it can be seen that any degree of introgression is only possible where there is no fitness cost associated with carrying the transgenic constructs. However, in contrast to the case with no lethal component, complete elimination of the transgenes is observed unless neither of the transgenic constructs confer any fitness cost (i.e. ${ɛ}_{A}=1={ɛ}_{B}$). This is due to the fact that when a lethal element is present, an individual must carry at least one copy of both constructs in order to survive. Thus, if one construct carried even a small fitness cost, then a selection pressure is created for that gene to be eliminated; in turn making individuals carrying the opposite transgenic construct unviable. It is expected that a small fitness cost could possibly be countered by utilising a larger release ratio to exceed the threshold transgene frequency. This appears to differ from cases with a 1:1 male-only release (Fig. 2(i) and (j)) which give no introgression even where no fitness cost is conferred by either transgenic construct. This is explored further in Section 3.1.1.

Finally, the results given in Fig. 2(b), (c), (g) and (h) demonstrate that a single 1:1 (bisex or male-only) release of individuals carrying strongly suppressed lethal elements (bisex or female-only) can give complete transgene introgression even in cases where carrying the constructs confers some fitness cost. The exact degree of fitness cost tolerable varies for each system. For example, a single 1:1 bisex release with strongly suppressed bisex lethal elements (Fig. 2(b)) is able to tolerate fitness costs in the region of  ∼ 12% (i.e. ε_*A*_ ≈ 0.88 ≈ ε_*B*_) whilst a male-only release of strongly suppressed female-only lethals (Fig. 2(h)) can tolerate just a  ∼ 5% fitness cost (i.e. ε_*A*_ ≈ 0.95 ≈ ε_*B*_).

This section has clearly demonstrated the differences in efficacy for various strategies and systems based on a 1:1 release ratio. In subsequent sections this is extended to consider a number of other factors important in both the design phase and in planning a release strategy for such a system.

#### 3.1.1 A single, male-only release with weak lethal suppression is ineffective

**Fig. 3:**
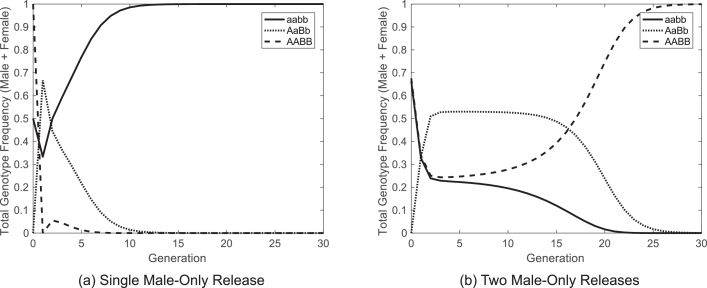
A single male-only release strategy with a weakly suppressed lethal cannot work. The examples shown are for a single (a) and two (b) male-only releases with 1:1 and 0.51:1 (introduced to wild-type) ratios, respectively (and relative fitnesses ${ɛ}_{A}=1={ɛ}_{B}$). Releases consist of individuals carrying weakly suppressed bisex lethals, however weakly suppressed female-only lethals give very similar results. In the single release case the first progeny produced are either transheterozygote (*AaBb*) or wild-type (*aabb*) since there are initially no transgenic females available for mating. The system therefore relies on transheterozygotes being able to exceed the release threshold which cannot happen for the release ratios considered in this work. If instead releases were made in the first two generations there will be double transgene homozygotes (*AABB*) available for mating with the initial progeny which allows the release threshold to be exceeded and thus transgenes to be introgressed.

It was shown in Fig. 2(i) and (j) that a strategy based on a single 1:1 male-only release of mosquitoes carrying weakly suppressed lethal elements (bisex or female-only) could not give any degree of long-term introgression. This section explores whether such systems merely require larger release ratios or whether there is some other factor leading to their failure. This begins by simulating the systems for single, male-only releases with a sequence of rising release ratios. In particular, these systems are simulated for a range of release ratios up to and including 10^6^:1 (introduced to wild-type) which is far beyond the range realistic for introduction in the field. Each simulation conducted here gave a final outcome whereby wild-type individuals return to fixation in the population in just a small number of generations (see for example Fig. 3(a)).

Results clearly indicate that the size of release is not the cause of failure in these systems and that some other factor must be at play. In order to assess what causes such a system to fail, a number of different factors including the number of releases were investigated. An example in which two male-only releases with a 0.51:1 ratio (introduced to total adults of all genotypes in the wild) are made in consecutive generations (with relative fitnesses ${ɛ}_{A}=1={ɛ}_{B}$) was considered (Fig. 3(b)). It can be seen here that the addition of a second release leads to lasting introgression of the introduced transgenes in spite of an extremely small increase in the overall number of insects released.

Based on these results it can be seen that the failure of such a system is due to a combination of the male-only release and the weak suppression of lethals. Following a single male-only release, all of the introduced double transgene homozygote (*AABB*) males must mate with wild-type (*aabb*) females. This differs from a bisex release in which introduced double transgene homozygote (*AABB*) males and females may mate one another, giving double homozygote (*AABB*) progeny which cannot occur in a male-only release strategy. The weak suppression of lethals does not make any difference to the genotype composition in the first generation after release. However, in the second generation post-release, a system with weakly suppressed lethals can only produce three genotypes (*aabb, AaBb* and *AABB*). In contrast to this, a strongly suppressed lethal system will allow two further genotypes to survive (*AABb* and *AaBB*), thereby increasing the transgene frequency in the population. Whilst either of these factors alone may be overcome by larger release ratios or efforts to engineer a system with smaller fitness costs, the combination results in a system that cannot produce a large enough transgene frequency in the population as to exceed the unstable equilibrium, thus resulting in transgene elimination. If instead, a two release (male-only) strategy is implemented, the second release introduces a new batch of double homozygote (*AABB*) males that can mate *aabb* and *AaBb* females produced in the initial generation. It appears that, depending on the release ratio considered, a second male-only release can be sufficient to allow a transgene frequency that exceeds the threshold for lasting introgression of the introduced transgenes.

### 3.2 Time to reach equilibrium

**Fig. 4:**
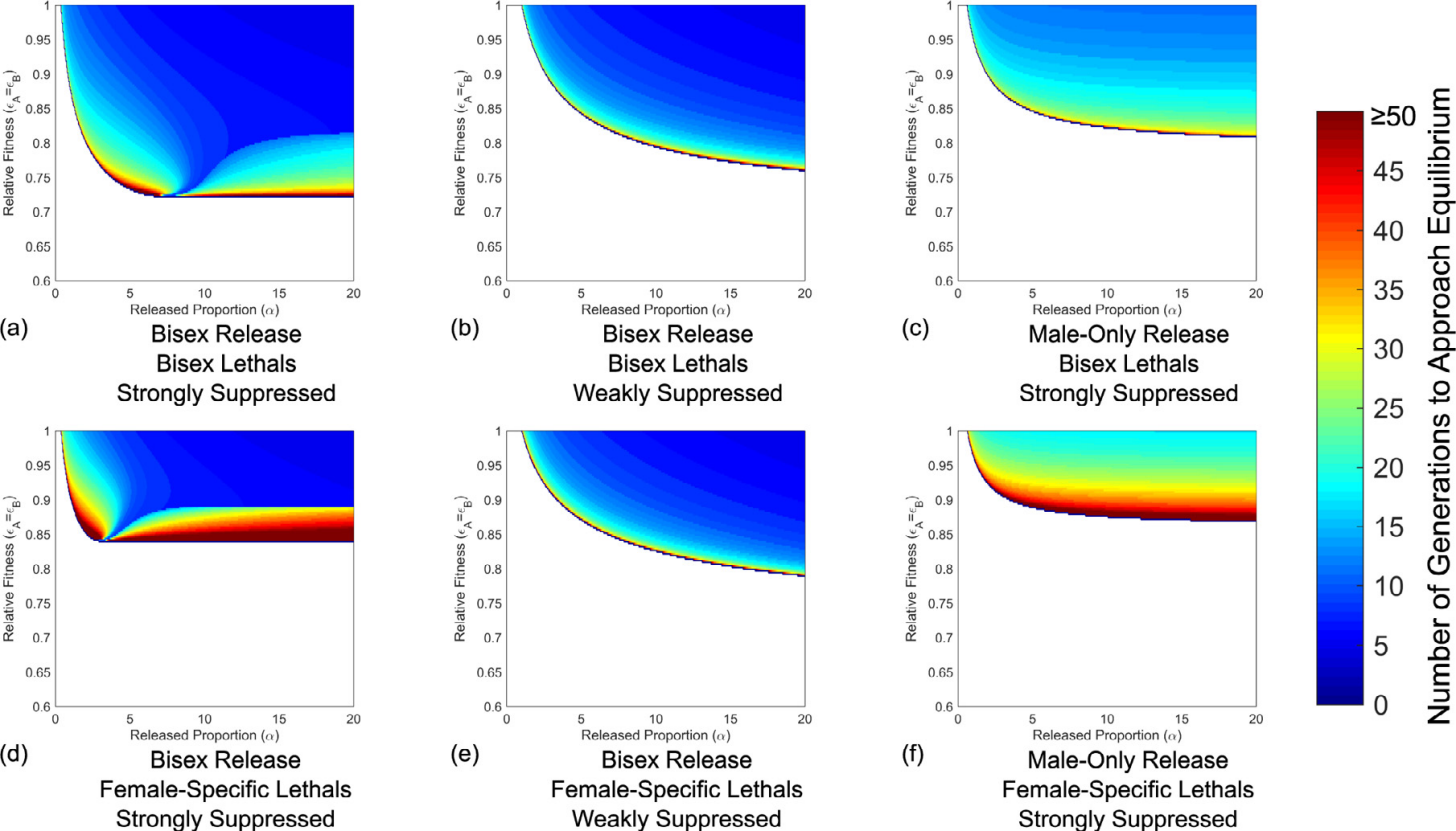
Different release strategies and genetic systems produce differences in the time taken to reach equilibrium. Plots show the time taken for the wild-type (*aabb*) genotype frequency to reach equilibrium. This is shown only for parameter spaces where transgene introgression is achieved. Here colours refer to the number of generations taken to reach equilibrium capped at 50 so as to show the detail of smaller numbers of generations. Strategies involving male-only releases of weakly suppressed lethal elements (both bisex and female-only) are not included here since they do not give any introgression for a single release strategy. Panels (b), (c), (e) and (f) appear to show a very similar pattern whilst panels (a) and (d) show a different pattern although there are differences in the location of features.

The ability of a system to ensure introgression of transgenes into a population and eliminate/reduce the prevalence of a pathogen is clearly the primary concern associated with an engineered underdominance control strategy. However, another key metric that has been suggested is that any control strategy considered for a field release should achieve its goal “on a human timescale” (Marshall and Hay, 2012); i.e. transgenes should be introgressed into an insect population and reduce the public health burden of infections in an observable time frame (James, 2005). In Section 3.1 it was shown that a number of different strategies could lead to the introgression of transgenes so long as they meet certain requirements on the conferred fitness costs for a single 1:1 (introduced to wild-type) release. Thus, in order to examine the time scale of action, the numbers of generations taken for wild-type (*aabb*) insects to be replaced by transgenic individuals for each strategy and genetic system considered are obtained. Male-only releases with weakly suppressed lethal elements are not considered here since they are not able to give any degree of introgression from a single release strategy. Results are obtained as per Section 2.3 and shown in Fig. 4.

From these results it is clear that when conditions for transgene introgression are met, this will tend to occur in under fifty generations. For the example of *Ae. aegypti* mosquitoes, this represents a time frame of less than five years; given at least 12 generations per year, which is realistic in tropical regions (Southwood, Murdie, Yasuno, Tonn, Reader, 1972, Tun-Lin, Burkot, Kay, 2000). There are however, regions of parameter space in which a two-locus engineered underdominance gene drive system would take fifty generations or longer to achieve introgression and these are all close to the threshold boundary.

It is evident here that bisex releases of weakly suppressed lethals (bisex or female-only) produce extremely similar patterns in terms of the number of generations taken to reach transgene introgression (Fig. 4(b) and (e)). In these cases, away from the threshold line the introgression of transgenes would occur in ten generations or less (under one year). A similar pattern is also displayed for male-only releases of strongly suppressed lethals (both bisex and female-only) as shown in Fig. 4(c) and (f). The main difference here is that for rapid transgene introgression, a strategy would be required to move further inside the threshold line since there is a larger region of parameter space in which introgression is slower. In spite of this, there is still a good range of parameter space that would lead to full transgene introgression in twenty five generations or less ( ∼ 2 years).

**Fig. 5:**
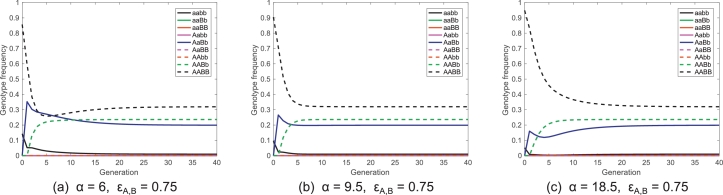
Plots showing examples of oscillation in different variables under various release ratios and fitness costs for a bisex release with strongly suppressed bisex lethals. Here panels demonstrate (a) an undershoot in the double transgene homozygote (*AABB*) genotype frequency; (b) no oscillation; and (c) oscillation in the transheterozygote (*AaBb*) genotype frequency. Each panel represents a particular point in parameter space from Fig. 4(a) with details of release ratios (*α*) and fitness costs (${ɛ}_{a}={ɛ}_{B}$) used given in the respective labels. Note that these three scenarios are located to the left, in the centre and to the right, respectively, of the divide in Fig. 4(a).

Finally, bisex releases with strongly suppressed lethals (both bisex and female-only) are shown in Fig. 4(a) and (d). These two strategies show a different pattern in that there are two distinct regions in which slow introgression is observed. This appears to be caused by a shift in oscillatory variable from *AABB* on the left of the divide to *AaBb* on the right (as seen in Fig. 5). Despite this, there is still a large region of parameter space in which complete introgression can be achieved in ten generations or less. It can also be seen that between the two regions of slower introgression there is a space in which low numbers of generations to introgression are observed in spite of relatively modest release ratios and fairly high fitness costs from carrying the transgenic constructs. Whether this region would be of use in real world release strategies however is doubtful as fitness costs and wild insect population sizes are both difficult to measure accurately, thus making it extremely hard to engineer a gene drive system capable of exploiting such a feature. Additionally, it can be seen in Fig. 5 that the wild-type (*aabb*) genotype frequency falls quickly in each case but takes a number of generations to settle to a final equilibrium where other genotype frequencies oscillate. Thus, it is unlikely that targeting these regions would produce a tangible benefit in terms of dengue reduction/elimination.

## 4 Multiple release strategies

**Fig. 6:**
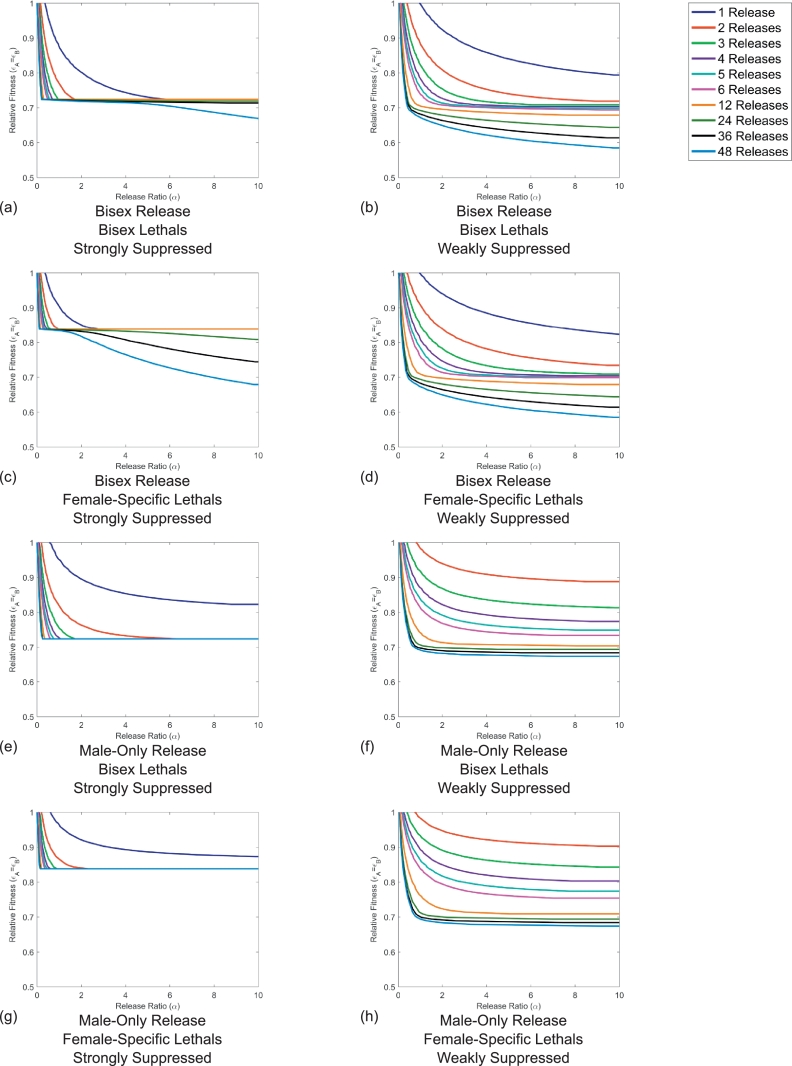
Different multiple release strategies and genetic systems produce different conditions for transgene introgression. Here each line represents the threshold above which introgression of transgenes into a population is achieved with colours representing different numbers of releases. Each panel represents a possible combination of bisex and male-only releases; bisex and female-only lethals; and strong or weak suppressors, with details given in figure labels. For strongly suppressed lethals there are significant differences between bisex and female-only lethals in terms of tolerable fitness costs. In all cases, bisex releases lead to greater tolerance of fitness costs as the release ratio is increased. Single male-only release of weakly suppressed lethals cannot give introgression even for very large release ratios, hence the omission of a single release threshold line in panels (f) and (h).

It was shown in Section 3.1.1 that for some types of systems, the implementation of more than one release can be essential for the success of that system (a necessary but not sufficient condition). As such, here it is investigated how strategies based on multiple releases of transgenic insects will affect the criteria for successful transgene introgression. In particular, using a similar strategy to that described in Section 2.2, the restrictions placed on release ratios and fitness costs in order to achieve transgene introgression with multiple releases are examined. Specifically, scenarios with ten different numbers (1, 2, 3, 4, 5, 6, 12, 24, 36, 48) of releases of equal size in consecutive generations are considered. These correspond to short control programs of up to  ∼ 6 months and longer programs of increasing length with approximately twelve month increments between each. Results of this are given in Fig. 6.

Fig. 6 shows threshold lines above which lasting introgression of the introduced transgenes is achieved. Below these threshold lines, wild-type individuals will out compete the introduced transgenic insects and thus return to fixation within the population. A more detailed view of low release ratio regions is given in Figure S1. Using these results it is possible to draw a number of conclusions that are useful when planning to test any such engineered underdominance gene drive system.

Firstly, it is clear that for a single release of transgenic insects, strongly suppressed lethals will be less restrictive in terms of the fitness costs that can be tolerated. However, this can be reversed when greater numbers of releases are added. For example, in Fig. 6(c) and (d) it can be seen that a six release strategy can tolerate fitness costs of up to  ∼ 30% in the weakly suppressed lethal case but only  ∼ 15% for the strongly suppressed case. The same general pattern holds for each of the strategies considered here except that in some cases, greater numbers of releases are required in order for this effect to become evident (see for example Fig. 6(e) and (f)).

It can also be seen that male-only releases are more restrictive than bisex releases in terms of the fitness costs tolerable while giving long term transgene introgression. However, in contrast to the case of strongly and weakly suppressed lethals, here larger release ratios and numbers of releases actually increase the difference between the two strategies. A similar observation was made by  Huang et al. (2009) who noted that for a single release, male-only strategies give higher introduction thresholds than bisex releases.

For now, consider just the examples with strongly suppressed lethals (i.e. Fig. 6(a), (c), (e) and (g)). Here it is clear that strategies built on bisex lethals can tolerate significantly larger fitness costs than those with female-only lethals. For example, in Fig. 6(e) it can be seen that a bisex lethal system can withstand fitness costs of  ∼ 27% whereas the female-only lethal system in Fig. 6(g) can only tolerate fitness costs of  ∼ 16%. This pattern is similar for Fig. 6(a) and (c) except that the effect is diminished for large numbers of high ratio releases.

For all strategies considered here there is a clear diminishing return from the addition of greater numbers of releases with release ratios up to *α* ≈ 1. For male-only releases with strongly suppressed lethals (Fig. 6(e) and (g)) it can be seen that above a release ratio of *α* ≈ 1 all of the threshold lines overlie each other. This suggests there is little benefit to making extra or larger ratio releases. Male-only releases with weakly suppressed lethals (both bisex and female-only) also display a diminishing return from adding greater numbers of releases. In particular, here the benefit in terms of increased tolerance to fitness costs appears to fall as more releases are added. Bisex releases (Fig. 6(a)–(d)) do not appear to display this diminishing return when adding extra releases of ratio *α* > 1. In cases with weakly suppressed lethals (Fig. 6(b) and (d)), for greater than twelve releases it appears that there is an approximately equal increase in tolerable fitness cost resulting from addition of extra releases. A similar effect can be seen for cases with strongly suppressed lethals (Fig. 6(a) and (c)) except that there is a region (*α* ∈ (1, 2)) in which the threshold lines approximately overlie one another. This would suggest that adding extra releases in this range would not be effective in increasing tolerable fitness costs.

## 5 Summary and discussion

We have formulated a population genetics model of two-locus engineered underdominance gene drive systems. This yields similar predictions to previous theoretical work in terms of introduction thresholds that give lasting transgene introgression from a single release ( ∼ 1/3 of the wild population for a system with strongly suppressed lethals and no fitness costs) (Davis et al., 2001). We also utilised this model to study different release strategies (number of releases and sexes released); lethal genes (bisex and female-specific); and strengths of lethal suppression (strong or weak). This revealed that for all genetic systems considered here it is possible to devise a release strategy that would give lasting transgene introgression so long as fitness costs fall into a tolerable range. We have also demonstrated that for feasible single release strategies, the equilibrium state is closely approached in fifty generations or less (under five years). In addition to this we studied the effects of adding extra releases for each strategy, demonstrating that bisex releases and weakly suppressed lethals are capable of tolerating greater fitness costs than male-only releases or strongly suppressed lethals, respectively. These results suggest that two-locus engineered underdominance gene drive represents a feasible strategy for replacement of insect populations and thus for the reduction/elimination of a number of insect vectored pathogens.

Previous work on engineered underdominance and other classes of gene drive systems have suggested a range of possible release strategies and genetic elements. Here we extend upon the previous literature by considering a range of different scenarios. Each of these appears feasible to engineer and have been shown here to possess different thresholds in terms of both the release ratios necessary to achieve lasting introgression and the degree of fitness cost that each system can tolerate.

The study of single release strategies showed that each of the systems considered may produce lasting transgene introgression under some conditions except for those based on male-only releases of individuals carrying weakly suppressed lethals. This was shown to be caused by two factors. In the first generation post-release, the male-only release strategy produces no *AABB* progeny, only *AaBb* and *aabb* since all introduced males must mate wild-type females. This has previously been seen as ideal in SIT and RIDL control since it would lead to the greatest degree of population suppression (Legros, Xu, Okamoto, Scott, Morrison, Lloyd, Gould, 2012, McInnis, Tam, Grace, Miyashita, 1994). In subsequent generations, weakly suppressed lethals cause only *AABB, AaBb* and *aabb* progeny to survive. This differs from strongly suppressed lethals that also allow *AABb* and *AaBB* progeny to survive. Each of these factors acts to reduce the transgene frequency in the population and in combination prevent such strategies from exceeding the transgene frequency required for lasting introgression.

For a single release of all feasible genetic systems considered here it was found that population replacement would tend to be achieved in fifty generations or less (under five years). Whilst this is the case, bisex releases generally took less time to achieve full introgression than did male-only releases. This is particularly interesting since male-only releases are likely to result in a more financially costly control programme since greater numbers of insects must be reared to obtain the desired number of males. A notable trait of all examples considered here is the existence of a region close to the threshold boundary where it would take a long time to achieve full introgression. This is due to the fact that these regions lie close to the unstable equilibrium of the system and thus are not initially as strongly attracted to the stable (full introgression) equilibrium as examples that begin further from this threshold boundary. However, any realistic strategy to be implemented in the field would likely require relative fitness parameters and release ratios to lie safely inside the threshold boundary in order to gain regulatory approval due to the uncertainty regarding exact numbers of insects within any given population; the limits of modelling assumptions (eg. demographics, stochasticity and genetic drift ignored); and biological features not accounted for (eg. previous generations preserved as dormant eggs). As such, it is anticipated that any realistic field-based release would achieve effective introgression of transgenes in less than five years.

Results given here also extend the previous theoretical literature through consideration of greater numbers of releases and an extended range of release ratios. These results clearly demonstrate the benefits resulting from the addition of greater numbers of repeated releases. In each case there is a clear benefit in terms of the tolerable fitness costs and release ratios that lead to lasting transgene introgression. Many of these cases do however display a diminishing return to the addition of extra releases. The extent of this diminishing return varies depending on the strategy considered and is most clearly seen in male-only releases. However, in spite of this diminishing return, it is likely that a strategy lying close to the borderline between success and failure could be improved either through engineering effort to reduce fitness costs; increasing the number of releases made; introducing insects at a greater release ratio; or some combination of these measures. These measures could also be used to provide a buffer against any uncertainty in the wild population size or measurements of fitness costs.

As with any mathematical model, this work is based on a number of simplifying assumptions that are common within this type of modelling work (Davis, Bax, Grewe, 2001, Magori, Gould, 2006, Marshall, Hay, 2012). Since the validity of these assumptions has been discussed previously they are not considered any further here. There are however a number of areas in which future work would be useful in order to better understand where engineered underdominance gene drive systems will be successful and where they will fail.

The genetic systems modelled here assume two distinct forms of lethal suppression. It is quite possible that a laboratory engineered system may not necessarily fit precisely into the categories of strong or weak lethal suppression, instead falling somewhere between these two classifications. As such, future work will be necessary in order to ascertain exactly how this affects the criteria for success of a given system. However, we would anticipate that release thresholds for such an intermediate system would lie somewhere between the two cases discussed here.

In this work a population genetics model based on discrete generations has been considered. For a species such as *Ae. aegypti* this assumption may be a reasonable approximation where populations are synchronised by climate conditions (e.g. wet and dry seasons), but is unlikely to hold for wild populations which are thought to reproduce continuously, at least in many areas (Southwood et al., 1972). To build upon this work it would be useful to reformulate this model as differential equations that would enable the consideration of population dynamics and timing of lethality, in a similar manner to that considered for other genetic control systems (Alphey, Bonsall, 2014, Phuc, Andreasen, Burton, Vass, Epton, Pape, Fu, Condon, Scaife, Donnelly, Coleman, White-Cooper, Alphey, 2007). It is feasible here that the timing of transgene lethality and/or density-dependent competition during the larval phase could alter the transgene introgression thresholds, however we would not expect to see large differences. It is also feasible that overlapping generations could allow a single, male-only release with weakly suppressed lethals to produce lasting transgene introgression if the released transgenic individuals survive long enough to mate with the first transgenic offspring.

Huang, Lloyd, Legros, Gould, 2009, Huang, Lloyd, Legros, Gould, 2011) have considered age and spatial structuring of mosquito populations in the context of two-locus engineered underdominance gene drive. This work could be extended to consider a number of the alternate release strategies and genetic systems studied here. This would enable us to examine the effects of different configurations of spatial release and how these may differ for the various systems considered within this study.

The model presented here could also be adapted into a two-deme version (such as those in Altrock, Traulsen, Reed, 2011, Altrock, Traulsen, Reeves, Reed, 2010, Marshall, Hay, 2012). This would allow the investigation of a number of factors important from a regulatory point of view. In particular it would allow for the identification of threshold migration rates that result in different outcomes. Here we would expect that high migration rates could result in one of two outcomes. They could potentially cause transgenes to spread into a neighbouring population, or alternatively the migration of wild-type individuals into the targeted population may reduce the transgene frequency below the necessary threshold thereby preventing successful transgene introgression. As shown by  Marshall and Hay (2012), for lower migration rates we would expect transgenes to spread within the target population and only reach extremely small frequencies in neighbouring populations.

Within this work it has been assumed that the cargo (refractory) genes are permanently linked to the transgenic constructs and that no resistance evolves within the population. The likelihood of these components becoming unlinked is currently unknown and may only be established in long term experiments, although the effects of this could be modelled in a manner similar to that of  Marshall (2008).

As discussed above, results here clearly demonstrate the thresholds that must be satisfied for successful transgene introgression and the timescale upon which this acts. This provides a clear indication that engineered underdominance gene drive systems could potentially be used to replace wild-populations with refractory individuals, thus reducing/eliminating the pathogen. However, the incidence of a pathogen cannot be directly related to transgene introgression levels without a number of additional modelling assumptions.

Due to the fairly general nature of modelling assumptions given here, we anticipate that results would be applicable to a range of insect species and pathogens. As discussed here, *Ae. aegypti* mosquitoes are the primary vectors of dengue. They are also vectors of yellow fever, chikungunya and Zika viruses. We thus anticipate that this model would be relevant for assessing the control of those viruses and other pathogens also. In addition to this, it is likely that results here would also be applicable to other mosquito-borne diseases such as malaria and filariasis, and other genetic pest management scenarios.

This study indicates that feasible release strategies and genetic systems are likely to allow successful transgene introgression across a range of fitness regimes. In particular, this work helps to define the relationship between specific genetic designs and constraints on appropriate release strategies. Further modelling work would likely be able to refine this further.

## Appendix A

A population genetics model of a two-locus engineered underdominance-based gene drive system is considered. This is based on a number of assumptions on the nature of the modelled population. Firstly, it is assumed that the population is sufficiently large that stochastic effects and integer numbers of individual mosquitoes may be neglected. It is then assumed that the modelled population is isolated (closed) and panmictic (randomly mating) with a 1:1 (male to female) sex ratio both initially and in each subsequent generation (although this may be skewed by the control programmes). Also, as discussed within the main text, it is assumed that the cargo (refractory gene) is perfectly linked to the suppressor genes (i.e. they can never become unlinked) and also that no resistance mechanism(s) are present or will evolve.

Through the introduction of two transgenic constructs into a wild population, a total of nine possible genotypes emerge. These are assigned a number (*i*) between one and nine and are denoted by the presence (*A* or *B*) or absence (*a* or *b*) of transgenic constructs *A* and *B*, respectively (for example, genotype 4 is *Aabb*, and related parameters have subscript 4). These are summarised in Table 1 whilst all variables and parameters are given in Table 2.

### Modelling fitness costs, lethals and suppressors

Within this model there are assumed to be two individual aspects contributing to the overall fitness of an individual relative to wild-type. The first of these relates to the fitness cost, i.e. a reduced survival resulting from the carrying of a transgene, independent of any lethal effect. This is implemented in the model by choosing appropriate values for the parameters ε_*A*_ and ε_*B*_ which represent the relative fitness (compared to wild-type) of an individual carrying a single copy of transgenic construct A or B, respectively. Here a value of ɛ=0 means an individual would be completely unviable whereas ɛ=1 represents an individual that is precisely as fit as wild-type. These fitness costs are applied multiplicatively for each construct such that individuals carrying two copies have a relative fitness of ε^2^. They are also applied multiplicatively across constructs so that an individual carrying two copies of each construct has a relative fitness of ${ɛ}_{A}^{2}{ɛ}_{B}^{2}$. It is also worth noting that an individual carrying no copies of, for example, construct A will have relative fitness ${ɛ}_{A}^{0}=1$.

Also considered here are the effects of lethal genetic components that are suppressed with varying effect by the suppressors contained on the construct at the other locus. As with fitness costs from carrying copies of the transgenes, the effects of these lethal and suppressing genetic elements are modelled through appropriate choices of parameter values *γ_i_* (i=1,…,9). Here a value of $\gamma_{i}=1$ represents a fully effective lethal (i.e. no individuals of the affected genotype survive) whereas $\gamma_{i}=0$ represents the absence of a lethal effect on a particular genotype. In the case of a strongly suppressed lethal, *γ_i_* are defined as

$$\gamma_{1}=\gamma_{5}=\gamma_{6}=\gamma_{8}=\gamma_{9}=0\;\;\text{and}\;\;\gamma_{2}=\gamma_{3}=\gamma_{4}=\gamma_{7}=1.$$

This differs from a weakly suppressed lethal for which we take

$$\gamma_{1}=\gamma_{5}=\gamma_{9}=0\;\;\text{and}\;\;\gamma_{2}=\gamma_{3}=\gamma_{4}=\gamma_{6}=\gamma_{7}=\gamma_{8}=1.$$

In the case of a bisex lethal these values are taken for both males ($\gamma_{i}^{M}$) and females ($\gamma_{i}^{F}$) whereas for a female-specific lethal, $\gamma_{i}^{M}=0$ whilst $\gamma_{i}^{F}$ are chosen as above.

Based on the chosen fitness costs and lethal effects discussed here it is possible to formulate an expression for the overall fitness of each genotype in terms of a fraction surviving to sexual maturity. This is given as

(1) $$\Omega_{i}^{M/F}={ɛ}_{A}^{\eta_{i}^{A}}{ɛ}_{B}^{\eta_{i}^{B}}\left(1-\gamma_{i}^{M/F}\right),$$

where $\eta_{i}^{A/B}$ denote the number of copies of transgenic construct A or B that are carried by a given genotype *i*.

### Modelling genotype frequencies

The assumptions stated above allow for the formulation of a population genetics model that is simulated in a two step manner. The first step is to calculate the proportion of the total population that will be of each genotype and sex. This is achieved by considering all possible matings between males and females of each genotype and the fraction of their respective progeny that will be of each genotype. These are scaled by $\Omega_{i}^{M/F}$ (Eq. (1)) representing the fraction of each sex (*M* or *F*, male or female) and genotype (*i*, see Table 1) surviving to sexual maturity. The proportion of individuals that are of each genotype may then be calculated using

(2) $$\begin{array}{ccc} M_{1}^{e} & = & \frac{\Omega_{1}^{M}}{2}(M_{1}^{t}F_{1}^{t}+\frac{1}{2}M_{1}^{t}F_{2}^{t}+\frac{1}{2}M_{1}^{t}F_{4}^{t}+\frac{1}{4}M_{1}^{t}F_{5}^{t}+\frac{1}{2}M_{2}^{t}F_{1}^{t}+\frac{1}{4}M_{2}^{t}F_{2}^{t}\\ & & +\;\frac{1}{4}M_{2}^{t}F_{4}^{t}+\frac{1}{8}M_{2}^{t}F_{5}^{t}+\frac{1}{2}M_{4}^{t}F_{1}^{t}+\frac{1}{4}M_{4}^{t}F_{2}^{t}+\frac{1}{4}M_{4}^{t}F_{4}^{t}+\frac{1}{8}M_{4}^{t}F_{5}^{t}\\ & & +\;\frac{1}{4}M_{5}^{t}F_{1}^{t}+\frac{1}{8}M_{5}^{t}F_{2}^{t}+\frac{1}{8}M_{5}^{t}F_{4}^{t}+\frac{1}{16}M_{5}^{t}F_{5}^{t}), \end{array}$$

(3) $$\begin{array}{ccc} M_{2}^{e} & = & \frac{\Omega_{2}^{M}}{2}(\frac{1}{2}M_{1}^{t}F_{2}^{t}+M_{1}^{t}F_{3}^{t}+\frac{1}{4}M_{1}^{t}F_{5}^{t}+\frac{1}{2}M_{1}^{t}F_{6}^{t}+\frac{1}{2}M_{2}^{t}F_{1}^{t}+\frac{1}{2}M_{2}^{t}F_{2}^{t}\\ & & +\;\frac{1}{2}M_{2}^{t}F_{3}^{t}+\frac{1}{4}M_{2}^{t}F_{4}^{t}+\frac{1}{4}M_{2}^{t}F_{5}^{t}+\frac{1}{4}M_{2}^{t}F_{6}^{t}+M_{3}^{t}F_{1}^{t}+\frac{1}{2}M_{3}^{t}F_{2}^{t}\\ & & +\;\frac{1}{2}M_{3}^{t}F_{4}^{t}+\frac{1}{4}M_{3}^{t}F_{5}^{t}+\frac{1}{4}M_{4}^{t}F_{2}^{t}+\frac{1}{2}M_{4}^{t}F_{3}^{t}+\frac{1}{8}M_{4}^{t}F_{5}^{t}+\frac{1}{4}M_{4}^{t}F_{6}^{t}\\ & & +\;\frac{1}{4}M_{5}^{t}F_{1}^{t}+\frac{1}{4}M_{5}^{t}F_{2}^{t}+\frac{1}{4}M_{5}^{t}F_{3}^{t}+\frac{1}{8}M_{5}^{t}F_{4}^{t}+\frac{1}{8}M_{5}^{t}F_{5}^{t}+\frac{1}{8}M_{5}^{t}F_{6}^{t}\\ & & +\;\frac{1}{2}M_{6}^{t}F_{1}^{t}+\frac{1}{4}M_{6}^{t}F_{2}^{t}+\frac{1}{4}M_{6}^{t}F_{4}^{t}+\frac{1}{8}M_{6}^{t}F_{5}^{t}), \end{array}$$

(4) $$\begin{array}{ccc} M_{3}^{e} & = & \frac{\Omega_{3}^{M}}{2}(\frac{1}{4}M_{2}^{t}F_{2}^{t}+\frac{1}{2}M_{2}^{t}F_{3}^{t}+\frac{1}{8}M_{2}^{t}F_{5}^{t}+\frac{1}{4}M_{2}^{t}F_{6}^{t}+\frac{1}{2}M_{3}^{t}F_{2}^{t}+M_{3}^{t}F_{3}^{t}\\ & & +\;\frac{1}{4}M_{3}^{t}F_{5}^{t}+\frac{1}{2}M_{3}^{t}F_{6}^{t}+\frac{1}{8}M_{5}^{t}F_{2}^{t}+\frac{1}{4}M_{5}^{t}F_{3}^{t}+\frac{1}{16}M_{5}^{t}F_{5}^{t}+\frac{1}{8}M_{5}^{t}F_{6}^{t}\\ & & +\;\frac{1}{4}M_{6}^{t}F_{2}^{t}+\frac{1}{2}M_{6}^{t}F_{3}^{t}+\frac{1}{8}M_{6}^{t}F_{5}^{t}+\frac{1}{4}M_{6}^{t}F_{6}^{t}), \end{array}$$

(5) $$\begin{array}{ccc} M_{4}^{e} & = & \frac{\Omega_{4}^{M}}{2}(\frac{1}{2}M_{1}^{t}F_{4}^{t}+\frac{1}{4}M_{1}^{t}F_{5}^{t}+M_{1}^{t}F_{7}^{t}+\frac{1}{2}M_{1}^{t}F_{8}^{t}+\frac{1}{4}M_{2}^{t}F_{4}^{t}+\frac{1}{8}M_{2}^{t}F_{5}^{t}\\ & & +\;\frac{1}{2}M_{2}^{t}F_{7}^{t}+\frac{1}{4}M_{2}^{t}F_{8}^{t}+\frac{1}{2}M_{4}^{t}F_{1}^{t}+\frac{1}{4}M_{4}^{t}F_{2}^{t}+\frac{1}{2}M_{4}^{t}F_{4}^{t}+\frac{1}{4}M_{4}^{t}F_{5}^{t}\\ & & +\;\frac{1}{2}M_{4}^{t}F_{7}^{t}+\frac{1}{4}M_{4}^{t}F_{8}^{t}+\frac{1}{4}M_{5}^{t}F_{1}^{t}+\frac{1}{8}M_{5}^{t}F_{2}^{t}+\frac{1}{4}M_{5}^{t}F_{4}^{t}\\ & & +\;\frac{1}{8}M_{5}^{t}F_{5}^{t}+\frac{1}{4}M_{5}^{t}F_{7}^{t}+\frac{1}{8}M_{5}^{t}F_{8}^{t}+M_{7}^{t}F_{1}^{t}+\frac{1}{2}M_{7}^{t}F_{2}^{t}+\frac{1}{2}M_{7}^{t}F_{4}^{t}\\ & & +\;\frac{1}{4}M_{7}^{t}F_{5}^{t}+\frac{1}{2}M_{8}^{t}F_{1}^{t}+\frac{1}{4}M_{8}^{t}F_{2}^{t}+\frac{1}{4}M_{8}^{t}F_{4}^{t}+\frac{1}{8}M_{8}^{t}F_{5}^{t}), \end{array}$$

(6) $$\begin{array}{ccc} M_{5}^{e} & = & \frac{\Omega_{5}^{M}}{2}(\frac{1}{4}M_{1}^{t}F_{5}^{t}+\frac{1}{2}M_{1}^{t}F_{6}^{t}+\frac{1}{2}M_{1}^{t}F_{8}^{t}+M_{1}^{t}F_{9}^{t}+\frac{1}{4}M_{2}^{t}F_{4}^{t}+\frac{1}{4}M_{2}^{t}F_{5}^{t}\\ & & +\;\frac{1}{4}M_{2}^{t}F_{6}^{t}+\frac{1}{2}M_{2}^{t}F_{7}^{t}+\frac{1}{2}M_{2}^{t}F_{8}^{t}+\frac{1}{2}M_{2}^{t}F_{9}^{t}+\frac{1}{2}M_{3}^{t}F_{4}^{t}+\frac{1}{4}M_{3}^{t}F_{5}^{t}\\ & & +\;M_{3}^{t}F_{7}^{t}+\frac{1}{2}M_{3}^{t}F_{8}^{t}+\frac{1}{4}M_{4}^{t}F_{2}^{t}+\frac{1}{2}M_{4}^{t}F_{3}^{t}+\frac{1}{4}M_{4}^{t}F_{5}^{t}+\frac{1}{2}M_{4}^{t}F_{6}^{t}\\ & & +\;\frac{1}{4}M_{4}^{t}F_{8}^{t}+\frac{1}{2}M_{4}^{t}F_{9}^{t}+\frac{1}{4}M_{5}^{t}F_{1}^{t}+\frac{1}{4}M_{5}^{t}F_{2}^{t}+\frac{1}{4}M_{5}^{t}F_{3}^{t}+\frac{1}{4}M_{5}^{t}F_{4}^{t}\\ & & +\;\frac{1}{4}M_{5}^{t}F_{5}^{t}+\frac{1}{4}M_{5}^{t}F_{6}^{t}+\frac{1}{4}M_{5}^{t}F_{7}^{t}+\frac{1}{4}M_{5}^{t}F_{8}^{t}+\frac{1}{4}M_{5}^{t}F_{9}^{t}+\frac{1}{2}M_{6}^{t}F_{1}^{t}\\ & & +\;\frac{1}{4}M_{6}^{t}F_{2}^{t}+\frac{1}{2}M_{6}^{t}F_{4}^{t}+\frac{1}{4}M_{6}^{t}F_{5}^{t}+\frac{1}{2}M_{6}^{t}F_{7}^{t}+\frac{1}{4}M_{6}^{t}F_{8}^{t}\\ & & +\;\frac{1}{2}M_{7}^{t}F_{2}^{t}+M_{7}^{t}F_{3}^{t}+\frac{1}{4}M_{7}^{t}F_{5}^{t}+\frac{1}{2}M_{7}^{t}F_{6}^{t}+\frac{1}{2}M_{8}^{t}F_{1}^{t}+\frac{1}{2}M_{8}^{t}F_{2}^{t}\\ & & +\;\frac{1}{2}M_{8}^{t}F_{3}^{t}+\frac{1}{4}M_{8}^{t}F_{4}^{t}+\frac{1}{4}M_{8}^{t}F_{5}^{t}+\frac{1}{4}M_{8}^{t}F_{6}^{t}+M_{9}^{t}F_{1}^{t}+\frac{1}{2}M_{9}^{t}F_{2}^{t}\\ & & +\;\frac{1}{2}M_{9}^{t}F_{4}^{t}+\frac{1}{4}M_{9}^{t}F_{5}^{t}), \end{array}$$

(7) $$\begin{array}{ccc} M_{6}^{e} & = & \frac{\Omega_{6}^{M}}{2}(\frac{1}{8}M_{2}^{t}F_{5}^{t}+\frac{1}{4}M_{2}^{t}F_{6}^{t}+\frac{1}{4}M_{2}^{t}F_{8}^{t}+\frac{1}{2}M_{2}^{t}F_{9}^{t}+\frac{1}{4}M_{3}^{t}F_{5}^{t}+\frac{1}{2}M_{3}^{t}F_{6}^{t}\\ & & +\;\frac{1}{2}M_{3}^{t}F_{8}^{t}+M_{3}^{t}F_{9}^{t}+\frac{1}{8}M_{5}^{t}F_{2}^{t}+\frac{1}{4}M_{5}^{t}F_{3}^{t}+\frac{1}{8}M_{5}^{t}F_{5}^{t}+\frac{1}{4}M_{5}^{t}F_{6}^{t}\\ & & +\;\frac{1}{8}M_{5}^{t}F_{8}^{t}+\frac{1}{4}M_{5}^{t}F_{9}^{t}+\frac{1}{4}M_{6}^{t}F_{2}^{t}+\frac{1}{2}M_{6}^{t}F_{3}^{t}+\frac{1}{4}M_{6}^{t}F_{5}^{t}+\frac{1}{2}M_{6}^{t}F_{6}^{t}\\ & & +\;\frac{1}{4}M_{6}^{t}F_{8}^{t}+\frac{1}{2}M_{6}^{t}F_{9}^{t}+\frac{1}{4}M_{8}^{t}F_{2}^{t}+\frac{1}{2}M_{8}^{t}F_{3}^{t}+\frac{1}{8}M_{8}^{t}F_{5}^{t}\\ & & +\;\frac{1}{4}M_{8}^{t}F_{6}^{t}+\frac{1}{2}M_{9}^{t}F_{2}^{t}+M_{9}^{t}F_{3}^{t}+\frac{1}{4}M_{9}^{t}F_{5}^{t}+\frac{1}{2}M_{9}^{t}F_{6}^{t}), \end{array}$$

(8) $$\begin{array}{ccc} M_{7}^{e} & = & \frac{\Omega_{7}^{M}}{2}(\frac{1}{4}M_{4}^{t}F_{4}^{t}+\frac{1}{8}M_{4}^{t}F_{5}^{t}+\frac{1}{2}M_{4}^{t}F_{7}^{t}+\frac{1}{4}M_{4}^{t}F_{8}^{t}+\frac{1}{8}M_{5}^{t}F_{4}^{t}\\ & & +\;\frac{1}{16}M_{5}^{t}F_{5}^{t}+\frac{1}{4}M_{5}^{t}F_{7}^{t}+\frac{1}{8}M_{5}^{t}F_{8}^{t}+\frac{1}{2}M_{7}^{t}F_{4}^{t}+\frac{1}{4}M_{7}^{t}F_{5}^{t}+M_{7}^{t}F_{7}^{t}\\ & & +\frac{1}{2}M_{7}^{t}F_{8}^{t}+\frac{1}{4}M_{8}^{t}F_{4}^{t}+\frac{1}{8}M_{8}^{t}F_{5}^{t}+\frac{1}{2}M_{8}^{t}F_{7}^{t}+\frac{1}{4}M_{8}^{t}F_{8}^{t}), \end{array}$$

(9) $$\begin{array}{ccc} M_{8}^{e} & = & \frac{\Omega_{8}^{M}}{2}(\frac{1}{8}M_{4}^{t}F_{5}^{t}+\frac{1}{4}M_{4}^{t}F_{6}^{t}+\frac{1}{4}M_{4}^{t}F_{8}^{t}+\frac{1}{2}M_{4}^{t}F_{9}^{t}+\frac{1}{8}M_{5}^{t}F_{4}^{t}+\frac{1}{8}M_{5}^{t}F_{5}^{t}\\ & & +\;\frac{1}{8}M_{5}^{t}F_{6}^{t}+\frac{1}{4}M_{5}^{t}F_{7}^{t}+\frac{1}{4}M_{5}^{t}F_{8}^{t}+\frac{1}{4}M_{5}^{t}F_{9}^{t}+\frac{1}{4}M_{6}^{t}F_{4}^{t}+\frac{1}{8}M_{6}^{t}F_{5}^{t}\\ & & +\;\frac{1}{2}M_{6}^{t}F_{7}^{t}+\frac{1}{4}M_{6}^{t}F_{8}^{t}+\frac{1}{4}M_{7}^{t}F_{5}^{t}+\frac{1}{2}M_{7}^{t}F_{6}^{t}+\frac{1}{2}M_{7}^{t}F_{8}^{t}+M_{7}^{t}F_{9}^{t}\\ & & +\frac{1}{4}M_{8}^{t}F_{4}^{t}+\frac{1}{4}M_{8}^{t}F_{5}^{t}+\frac{1}{4}M_{8}^{t}F_{6}^{t}+\frac{1}{2}M_{8}^{t}F_{7}^{t}+\frac{1}{2}M_{8}^{t}F_{8}^{t}\\ & & +\;\frac{1}{2}M_{8}^{t}F_{9}^{t}+\frac{1}{2}M_{9}^{t}F_{4}^{t}+\frac{1}{4}M_{9}^{t}F_{5}^{t}+M_{9}^{t}F_{7}^{t}+\frac{1}{2}M_{9}^{t}F_{8}^{t}), \end{array}$$

(10) $$\begin{array}{ccc} M_{9}^{e} & = & \frac{\Omega_{9}^{M}}{2}(\frac{1}{16}M_{5}^{t}F_{5}^{t}+\frac{1}{8}M_{5}^{t}F_{6}^{t}+\frac{1}{8}M_{5}^{t}F_{8}^{t}+\frac{1}{4}M_{5}^{t}F_{9}^{t}+\frac{1}{8}M_{6}^{t}F_{5}^{t}\\ & & +\;\frac{1}{4}M_{6}^{t}F_{6}^{t}+\frac{1}{4}M_{6}^{t}F_{8}^{t}+\frac{1}{2}M_{6}^{t}F_{9}^{t}+\frac{1}{8}M_{8}^{t}F_{5}^{t}+\frac{1}{4}M_{8}^{t}F_{6}^{t}+\frac{1}{4}M_{8}^{t}F_{8}^{t}\\ & & +\;\frac{1}{2}M_{8}^{t}F_{9}^{t}+\frac{1}{4}M_{9}^{t}F_{5}^{t}+\frac{1}{2}M_{9}^{t}F_{6}^{t}+\frac{1}{2}M_{9}^{t}F_{8}^{t}+M_{9}^{t}F_{9}^{t}), \end{array}$$

(11) $$\begin{array}{ccc} F_{1}^{e} & = & \frac{\Omega_{1}^{F}}{2}(F_{1}^{t}M_{1}^{t}+\frac{1}{2}F_{1}^{t}M_{2}^{t}+\frac{1}{2}F_{1}^{t}M_{4}^{t}+\frac{1}{4}F_{1}^{t}M_{5}^{t}+\frac{1}{2}F_{2}^{t}M_{1}^{t}+\frac{1}{4}F_{2}^{t}M_{2}^{t}\\ & & +\;\frac{1}{4}F_{2}^{t}M_{4}^{t}+\frac{1}{8}F_{2}^{t}M_{5}^{t}+\frac{1}{2}F_{4}^{t}M_{1}^{t}+\frac{1}{4}F_{4}^{t}M_{2}^{t}+\frac{1}{4}F_{4}^{t}M_{4}^{t}\\ & & +\;\frac{1}{8}F_{4}^{t}M_{5}^{t}+\frac{1}{4}F_{5}^{t}M_{1}^{t}+\frac{1}{8}F_{5}^{t}M_{2}^{t}+\frac{1}{8}F_{5}^{t}M_{4}^{t}+\frac{1}{16}F_{5}^{t}M_{5}^{t}), \end{array}$$

(12) $$\begin{array}{ccc} F_{2}^{e} & = & \frac{\Omega_{2}^{F}}{2}(\frac{1}{2}F_{1}^{t}M_{2}^{t}+F_{1}^{t}M_{3}^{t}+\frac{1}{4}F_{1}^{t}M_{5}^{t}+\frac{1}{2}F_{1}^{t}M_{6}^{t}+\frac{1}{2}F_{2}^{t}M_{1}^{t}+\frac{1}{2}F_{2}^{t}M_{2}^{t}\\ & & +\;\frac{1}{2}F_{2}^{t}M_{3}^{t}+\frac{1}{4}F_{2}^{t}M_{4}^{t}+\frac{1}{4}F_{2}^{t}M_{5}^{t}+\frac{1}{4}F_{2}^{t}M_{6}^{t}+F_{3}^{t}M_{1}^{t}+\frac{1}{2}F_{3}^{t}M_{2}^{t}\\ & & +\;\frac{1}{2}F_{3}^{t}M_{4}^{t}+\frac{1}{4}F_{3}^{t}M_{5}^{t}+\frac{1}{4}F_{4}^{t}M_{2}^{t}+\frac{1}{2}F_{4}^{t}M_{3}^{t}+\frac{1}{8}F_{4}^{t}M_{5}^{t}+\frac{1}{4}F_{4}^{t}M_{6}^{t}\\ & & +\;\frac{1}{4}F_{5}^{t}M_{1}^{t}+\frac{1}{4}F_{5}^{t}M_{2}^{t}+\frac{1}{4}F_{5}^{t}M_{3}^{t}+\frac{1}{8}F_{5}^{t}M_{4}^{t}+\frac{1}{8}F_{5}^{t}M_{5}^{t}\\ & & +\;\frac{1}{8}F_{5}^{t}M_{6}^{t}+\frac{1}{2}F_{6}^{t}M_{1}^{t}+\frac{1}{4}F_{6}^{t}M_{2}^{t}+\frac{1}{4}F_{6}^{t}M_{4}^{t}+\frac{1}{8}F_{6}^{t}M_{5}^{t}), \end{array}$$

(13) $$\begin{array}{ccc} F_{3}^{e} & = & \frac{\Omega_{3}^{F}}{2}(\frac{1}{4}F_{2}^{t}M_{2}^{t}+\frac{1}{2}F_{2}^{t}M_{3}^{t}+\frac{1}{8}F_{2}^{t}M_{5}^{t}+\frac{1}{4}F_{2}^{t}M_{6}^{t}+\frac{1}{2}F_{3}^{t}M_{2}^{t}+F_{3}^{t}M_{3}^{t}\\ & & +\;\frac{1}{4}F_{3}^{t}M_{5}^{t}+\frac{1}{2}F_{3}^{t}M_{6}^{t}+\frac{1}{8}F_{5}^{t}M_{2}^{t}+\frac{1}{4}F_{5}^{t}M_{3}^{t}+\frac{1}{16}F_{5}^{t}M_{5}^{t}\\ & & +\;\frac{1}{8}F_{5}^{t}M_{6}^{t}+\frac{1}{4}F_{6}^{t}M_{2}^{t}+\frac{1}{2}F_{6}^{t}M_{3}^{t}+\frac{1}{8}F_{6}^{t}M_{5}^{t}+\frac{1}{4}F_{6}^{t}M_{6}^{t}), \end{array}$$

(14) $$\begin{array}{ccc} F_{4}^{e} & = & \frac{\Omega_{4}^{F}}{2}(\frac{1}{2}F_{1}^{t}M_{4}^{t}+\frac{1}{4}F_{1}^{t}M_{5}^{t}+F_{1}^{t}M_{7}^{t}+\frac{1}{2}F_{1}^{t}M_{8}^{t}+\frac{1}{4}F_{2}^{t}M_{4}^{t}+\frac{1}{8}F_{2}^{t}M_{5}^{t}\\ & & +\;\frac{1}{2}F_{2}^{t}M_{7}^{t}+\frac{1}{4}F_{2}^{t}M_{8}^{t}+\frac{1}{2}F_{4}^{t}M_{1}^{t}+\frac{1}{4}F_{4}^{t}M_{2}^{t}+\frac{1}{2}F_{4}^{t}M_{4}^{t}\\ & & +\;\frac{1}{4}F_{4}^{t}M_{5}^{t}+\frac{1}{2}F_{4}^{t}M_{7}^{t}+\frac{1}{4}F_{4}^{t}M_{8}^{t}+\frac{1}{4}F_{5}^{t}M_{1}^{t}+\frac{1}{8}F_{5}^{t}M_{2}^{t}\\ & & +\;\frac{1}{4}F_{5}^{t}M_{4}^{t}+\frac{1}{8}F_{5}^{t}M_{5}^{t}+\frac{1}{4}F_{5}^{t}M_{7}^{t}+\frac{1}{8}F_{5}^{t}M_{8}^{t}+F_{7}^{t}M_{1}^{t}+\frac{1}{2}F_{7}^{t}M_{2}^{t}\\ & & +\;\frac{1}{2}F_{7}^{t}M_{4}^{t}+\frac{1}{4}F_{7}^{t}M_{5}^{t}+\frac{1}{2}F_{8}^{t}M_{1}^{t}+\frac{1}{4}F_{8}^{t}M_{2}^{t}+\frac{1}{4}F_{8}^{t}M_{4}^{t}\\ & & +\;\frac{1}{8}F_{8}^{t}M_{5}^{t}), \end{array}$$

(15) $$\begin{array}{ccc} F_{5}^{e} & = & \frac{\Omega_{5}^{F}}{2}(\frac{1}{4}F_{1}^{t}M_{5}^{t}+\frac{1}{2}F_{1}^{t}M_{6}^{t}+\frac{1}{2}F_{1}^{t}M_{8}^{t}+F_{1}^{t}M_{9}^{t}+\frac{1}{4}F_{2}^{t}M_{4}^{t}+\frac{1}{4}F_{2}^{t}M_{5}^{t}\\ & & +\;\frac{1}{4}F_{2}^{t}M_{6}^{t}+\frac{1}{2}F_{2}^{t}M_{7}^{t}+\frac{1}{2}F_{2}^{t}M_{8}^{t}+\frac{1}{2}F_{2}^{t}M_{9}^{t}+\frac{1}{2}F_{3}^{t}M_{4}^{t}\\ & & +\;\frac{1}{4}F_{3}^{t}M_{5}^{t}+F_{3}^{t}M_{7}^{t}+\frac{1}{2}F_{3}^{t}M_{8}^{t}+\frac{1}{4}F_{4}^{t}M_{2}^{t}+\frac{1}{2}F_{4}^{t}M_{3}^{t}+\frac{1}{4}F_{4}^{t}M_{5}^{t}\\ & & +\;\frac{1}{2}F_{4}^{t}M_{6}^{t}+\frac{1}{4}F_{4}^{t}M_{8}^{t}+\frac{1}{2}F_{4}^{t}M_{9}^{t}+\frac{1}{4}F_{5}^{t}M_{1}^{t}+\frac{1}{4}F_{5}^{t}M_{2}^{t}\\ & & +\;\frac{1}{4}F_{5}^{t}M_{3}^{t}+\frac{1}{4}F_{5}^{t}M_{4}^{t}+\frac{1}{4}F_{5}^{t}M_{5}^{t}+\frac{1}{4}F_{5}^{t}M_{6}^{t}+\frac{1}{4}F_{5}^{t}M_{7}^{t}\\ & & +\;\frac{1}{4}F_{5}^{t}M_{8}^{t}+\frac{1}{4}F_{5}^{t}M_{9}^{t}+\frac{1}{2}F_{6}^{t}M_{1}^{t}+\frac{1}{4}F_{6}^{t}M_{2}^{t}+\frac{1}{2}F_{6}^{t}M_{4}^{t}\\ & & +\;\frac{1}{4}F_{6}^{t}M_{5}^{t}+\frac{1}{2}F_{6}^{t}M_{7}^{t}+\frac{1}{4}F_{6}^{t}M_{8}^{t}+\frac{1}{2}F_{7}^{t}M_{2}^{t}+F_{7}^{t}M_{3}^{t}+\frac{1}{4}F_{7}^{t}M_{5}^{t}\\ & & +\;\frac{1}{2}F_{7}^{t}M_{6}^{t}+\frac{1}{2}F_{8}^{t}M_{1}^{t}+\frac{1}{2}F_{8}^{t}M_{2}^{t}+\frac{1}{2}F_{8}^{t}M_{3}^{t}+\frac{1}{4}F_{8}^{t}M_{4}^{t}\\ & & +\;\frac{1}{4}F_{8}^{t}M_{5}^{t}+\frac{1}{4}F_{8}^{t}M_{6}^{t}+F_{9}^{t}M_{1}^{t}+\frac{1}{2}F_{9}^{t}M_{2}^{t}+\frac{1}{2}F_{9}^{t}M_{4}^{t}+\frac{1}{4}F_{9}^{t}M_{5}^{t}), \end{array}$$

(16) $$\begin{array}{ccc} F_{6}^{e} & = & \frac{\Omega_{6}^{F}}{2}(\frac{1}{8}F_{2}^{t}M_{5}^{t}+\frac{1}{4}F_{2}^{t}M_{6}^{t}+\frac{1}{4}F_{2}^{t}M_{8}^{t}+\frac{1}{2}F_{2}^{t}M_{9}^{t}+\frac{1}{4}F_{3}^{t}M_{5}^{t}\\ & & +\frac{1}{2}F_{3}^{t}M_{6}^{t}+\frac{1}{2}F_{3}^{t}M_{8}^{t}+F_{3}^{t}M_{9}^{t}+\frac{1}{8}F_{5}^{t}M_{2}^{t}+\frac{1}{4}F_{5}^{t}M_{3}^{t}+\frac{1}{8}F_{5}^{t}M_{5}^{t}\\ & & +\frac{1}{4}F_{5}^{t}M_{6}^{t}+\frac{1}{8}F_{5}^{t}M_{8}^{t}+\frac{1}{4}F_{5}^{t}M_{9}^{t}+\frac{1}{4}F_{6}^{t}M_{2}^{t}+\frac{1}{2}F_{6}^{t}M_{3}^{t}+\frac{1}{4}F_{6}^{t}M_{5}^{t}\\ & & +\frac{1}{2}F_{6}^{t}M_{6}^{t}+\frac{1}{4}F_{6}^{t}M_{8}^{t}+\frac{1}{2}F_{6}^{t}M_{9}^{t}+\frac{1}{4}F_{8}^{t}M_{2}^{t}+\frac{1}{2}F_{8}^{t}M_{3}^{t}\\ & & +\frac{1}{8}F_{8}^{t}M_{5}^{t}+\frac{1}{4}F_{8}^{t}M_{6}^{t}+\frac{1}{2}F_{9}^{t}M_{2}^{t}+F_{9}^{t}M_{3}^{t}+\frac{1}{4}F_{9}^{t}M_{5}^{t}+\frac{1}{2}F_{9}^{t}M_{6}^{t}), \end{array}$$

(17) $$\begin{array}{ccc} F_{7}^{e} & = & \frac{\Omega_{7}^{F}}{2}(\frac{1}{4}F_{4}^{t}M_{4}^{t}+\frac{1}{8}F_{4}^{t}M_{5}^{t}+\frac{1}{2}F_{4}^{t}M_{7}^{t}+\frac{1}{4}F_{4}^{t}M_{8}^{t}+\frac{1}{8}F_{5}^{t}M_{4}^{t}\\ & & +\frac{1}{16}F_{5}^{t}M_{5}^{t}+\frac{1}{4}F_{5}^{t}M_{7}^{t}+\frac{1}{8}F_{5}^{t}M_{8}^{t}+\frac{1}{2}F_{7}^{t}M_{4}^{t}+\frac{1}{4}F_{7}^{t}M_{5}^{t}+F_{7}^{t}M_{7}^{t}\\ & & +\;\frac{1}{2}F_{7}^{t}M_{8}^{t}+\frac{1}{4}F_{8}^{t}M_{4}^{t}+\frac{1}{8}F_{8}^{t}M_{5}^{t}+\frac{1}{2}F_{8}^{t}M_{7}^{t}+\frac{1}{4}F_{8}^{t}M_{8}^{t}), \end{array}$$

(18) $$\begin{array}{ccc} F_{8}^{e} & = & \frac{\Omega_{8}^{F}}{2}(\frac{1}{8}F_{4}^{t}M_{5}^{t}+\frac{1}{4}F_{4}^{t}M_{6}^{t}+\frac{1}{4}F_{4}^{t}M_{8}^{t}+\frac{1}{2}F_{4}^{t}M_{9}^{t}+\frac{1}{8}F_{5}^{t}M_{4}^{t}\\ & & +\frac{1}{8}F_{5}^{t}M_{5}^{t}+\frac{1}{8}F_{5}^{t}M_{6}^{t}+\frac{1}{4}F_{5}^{t}M_{7}^{t}+\frac{1}{4}F_{5}^{t}M_{8}^{t}+\frac{1}{4}F_{5}^{t}M_{9}^{t}+\frac{1}{4}F_{6}^{t}M_{4}^{t}\\ & & +\frac{1}{8}F_{6}^{t}M_{5}^{t}+\frac{1}{2}F_{6}^{t}M_{7}^{t}+\frac{1}{4}F_{6}^{t}M_{8}^{t}+\frac{1}{4}F_{7}^{t}M_{5}^{t}+\frac{1}{2}F_{7}^{t}M_{6}^{t}+\frac{1}{2}F_{7}^{t}M_{8}^{t}\\ & & +\;F_{7}^{t}M_{9}^{t}+\frac{1}{4}F_{8}^{t}M_{4}^{t}+\frac{1}{4}F_{8}^{t}M_{5}^{t}+\frac{1}{4}F_{8}^{t}M_{6}^{t}+\frac{1}{2}F_{8}^{t}M_{7}^{t}+\frac{1}{2}F_{8}^{t}M_{8}^{t}\\ & & +\frac{1}{2}F_{8}^{t}M_{9}^{t}+\frac{1}{2}F_{9}^{t}M_{4}^{t}+\frac{1}{4}F_{9}^{t}M_{5}^{t}+F_{9}^{t}M_{7}^{t}+\frac{1}{2}F_{9}^{t}M_{8}^{t}), \end{array}$$

(19) $$\begin{array}{ccc} F_{9}^{e} & = & \frac{\Omega_{9}^{F}}{2}(\frac{1}{16}F_{5}^{t}M_{5}^{t}+\frac{1}{8}F_{5}^{t}M_{6}^{t}+\frac{1}{8}F_{5}^{t}M_{8}^{t}+\frac{1}{4}F_{5}^{t}M_{9}^{t}+\frac{1}{8}F_{6}^{t}M_{5}^{t}\\ & & +\frac{1}{4}F_{6}^{t}M_{6}^{t}+\frac{1}{4}F_{6}^{t}M_{8}^{t})\\ & & +\;\frac{1}{2}F_{6}^{t}M_{9}^{t}+\frac{1}{8}F_{8}^{t}M_{5}^{t}+\frac{1}{4}F_{8}^{t}M_{6}^{t}+\frac{1}{4}F_{8}^{t}M_{8}^{t}+\frac{1}{2}F_{8}^{t}M_{9}^{t}+\frac{1}{4}F_{9}^{t}M_{5}^{t}\\ & & +\;\frac{1}{2}F_{9}^{t}M_{6}^{t}+\frac{1}{2}F_{9}^{t}M_{8}^{t}+F_{9}^{t}M_{9}^{t}). \end{array}$$

Step two in this process is to normalise these proportions such that they fill the range zero to one. Thus, the genotype frequencies are obtained using

(20) $$\begin{array}{ccc} M_{1}^{t+1} & = & \frac{M_{1}^{e}}{\bar{\Omega }},\;\;\;M_{2}^{t+1}=\frac{M_{2}^{e}}{\bar{\Omega }},\;\;\;M_{3}^{t+1}=\frac{M_{3}^{e}}{\bar{\Omega }},\;\;\;M_{4}^{t+1}=\frac{M_{4}^{e}}{\bar{\Omega }},\\ M_{5}^{t+1} & = & \frac{M_{5}^{e}}{\bar{\Omega }},\;\;\;M_{6}^{t+1}=\frac{M_{6}^{e}}{\bar{\Omega }},\;\;\;M_{7}^{t+1}=\frac{M_{7}^{e}}{\bar{\Omega }},\\ M_{8}^{t+1} & = & \frac{M_{8}^{e}}{\bar{\Omega }},\;\;\;M_{9}^{t+1}=\frac{M_{9}^{e}}{\bar{\Omega }},\;\;\;F_{1}^{t+1}=\frac{F_{1}^{e}}{\bar{\Omega }},\\ F_{2}^{t+1} & = & \frac{F_{2}^{e}}{\bar{\Omega }},\;\;\;F_{3}^{t+1}=\frac{F_{3}^{e}}{\bar{\Omega }},\;\;\;F_{4}^{t+1}=\frac{F_{4}^{e}}{\bar{\Omega }},\\ F_{5}^{t+1} & = & \frac{F_{5}^{e}}{\bar{\Omega }},\;\;\;F_{6}^{t+1}=\frac{F_{6}^{e}}{\bar{\Omega }},\;\;\;F_{7}^{t+1}=\frac{F_{7}^{e}}{\bar{\Omega }},\\ F_{8}^{t+1} & = & \frac{F_{8}^{e}}{\bar{\Omega }},\;\;\;F_{9}^{t+1}=\frac{F_{9}^{e}}{\bar{\Omega }}, \end{array}$$

where the normalising factor $\bar{\Omega }$ is the sum of all proportional frequencies and is given by

(21) $$\bar{\Omega }=\sum_{i=1}^{9}\left(M_{i}^{e}+F_{i}^{e}\right).$$

### Modelling release strategies

In the main text 1:1 (introduced to wild-type) releases are considered before progressing on to the consideration of a wider range of release ratios and numbers of releases. For the case of a single release strategy, this is implemented via an appropriate choice of initial conditions. For a single bisex release we choose initial conditions of the form

$$\begin{array}{ccc} & & M_{1}^{0}=\frac{0.5}{1+\alpha },\;\;\;M_{9}^{0}=\frac{\alpha /2}{1+\alpha },\;\;\;F_{1}^{0}=\frac{0.5}{1+\alpha },\;\;\;F_{9}^{0}=\frac{\alpha /2}{1+\alpha }\\ & & \text{and}\;\;\;M_{2-8}^{0}=0=F_{2-8}^{0}, \end{array}$$

where the release ratio is defined as α=introduced/wild-type. This differs from a single male-only release for which initial conditions of the form

$$M_{1}^{0}=\frac{0.5}{1+\alpha },\;\;\;M_{9}^{0}=\frac{\alpha }{1+\alpha },\;\;F_{1}^{0}=\frac{0.5}{1+\alpha }\;\;\;\text{and}\;\;\;M_{2-8}^{0}=0=F_{2-9}^{0},$$

are considered.

Also considered here are scenarios in which a strategy consists of multiple releases. This requires the choice of both appropriate initial conditions and a suitable normalisation procedure. The first release of individuals is achieved by considering the initial conditions given above. In each subsequent time-step the re-normalisation process from (20) is altered in order to represent the addition of more double homozygote transgenic individuals at a ratio (*α*) defined in terms of the total population in the wild. For bisex releases this normalisation process takes the form

$$\begin{array}{ccc} & & M_{i}^{t+1}=\frac{M_{i}^{e}}{\left(1+\alpha \right)\bar{\Omega }},\;\;\;F_{i}^{t+1}=\frac{F_{i}^{e}}{\left(1+\alpha \right)\bar{\Omega }},\;\;\;M_{9}^{t+1}=\frac{M_{9}^{e}+\bar{\Omega }\alpha /2}{\left(1+\alpha \right)\bar{\Omega }},\\ & & \text{and}\;\;\;F_{9}^{t+1}=\frac{F_{9}^{e}+\bar{\Omega }\alpha /2}{\left(1+\alpha \right)\bar{\Omega }}, \end{array}$$

where i=1,…,8. This is similar to the normalisation procedure for male-only releases which is of the form

$$M_{i}^{t+1}=\frac{M_{i}^{e}}{\left(1+\alpha \right)\bar{\Omega }},\;\;\;F_{j}^{t+1}=\frac{F_{j}^{e}}{\left(1+\alpha \right)\bar{\Omega }},\;\;\text{and}\;\;M_{9}^{t+1}=\frac{M_{9}^{e}+\bar{\Omega }\alpha }{\left(1+\alpha \right)\bar{\Omega }},$$

where i=1,…,8 and j=1,…,9. These normalisation procedures are used for each generation in which transgenic insects are released. Once releases end, the model reverts back to the procedure given in (20) for all remaining generations.

## Appendix B Supplementary materials

Supplementary Data S1 Supplementary Raw Research Data. This is open data under the CC BY license http://creativecommons.org/licenses/by/4.0/ Supplementary Data S1
