# Supplementary material for: Conditions for success of engineered underdominance gene drive systems
Source: J Theor Biol. 2017 Oct 7;430:128–40. doi: 10.1016/j.jtbi.2017.07.014 (PMC5562440; doi:10.1016/j.jtbi.2017.07.014)
Supplement: Supplementary Data S1 — Supplementary Raw Research Data. This is open data under the CC BY license http://creativecommons.org/licenses/by/4.0/ [file mmc1.pdf]

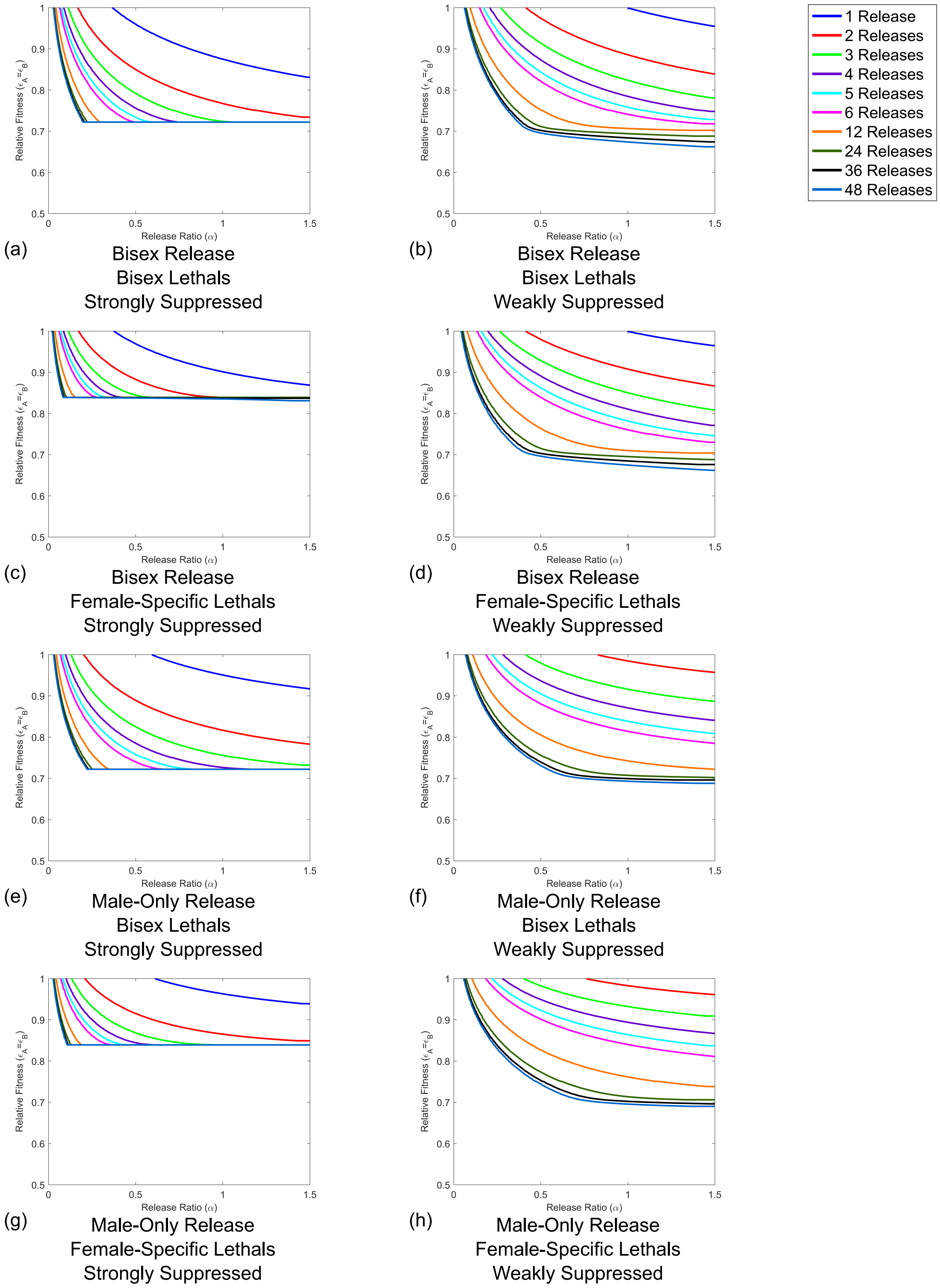

Figure S1: **Different multiple release strategies and genetic systems produce different conditions for transgene introgression.** Here each panel represents a more detailed view of the low release ratio regions of Figure 6 in the main text. Each line represents the threshold above which introgression of transgenes into a population is achieved with colours representing different numbers of releases. Details of the scenario considered in each panel are given in the respective labels. Single male-only release of weakly suppressed lethals cannot give introgression even for very large release ratios, hence the omission of a single release threshold line in panels (f) and (h).
